# The first complete mitochondrial genome of *Loimia arborea* (Polychaeta: Terebellidae) and phylogenetic analysis

**DOI:** 10.1080/23802359.2024.2429639

**Published:** 2024-11-25

**Authors:** Xiong-hui Xu, Chao-yang Luo, Yuan Mu

**Affiliations:** Institute of Eastern-Himalaya Biodiversity Research, Dali University, Dali, China

**Keywords:** *Loimia arborea*, mitochondrial genome, Terebellida, phylogenetic analysis, *trnM* duplication

## Abstract

In order to understand the molecular insights within the Terebellida, the complete mitochondrial genome of Loimia arborea was sequenced. The mitochondrial genome is 16,023 bp, with 13 protein-coding genes (PCGs), 23 transfer RNA (tRNA) genes, two ribosomal RNA (rRNA) genes, and a non-coding region (D-loop). Notably, two adjacent copies of methionine tRNA genes (trnMs) were detected. The phylogeny of Terebellida was constructed based on 13 PCGs from 13 species, two main clades were strongly supported, i.e., Cirratuliformia (clade A) and Terebelliformia (clade B). And the monophyly of the Terebellidae was restored.

## Introduction

**Figure 1.**
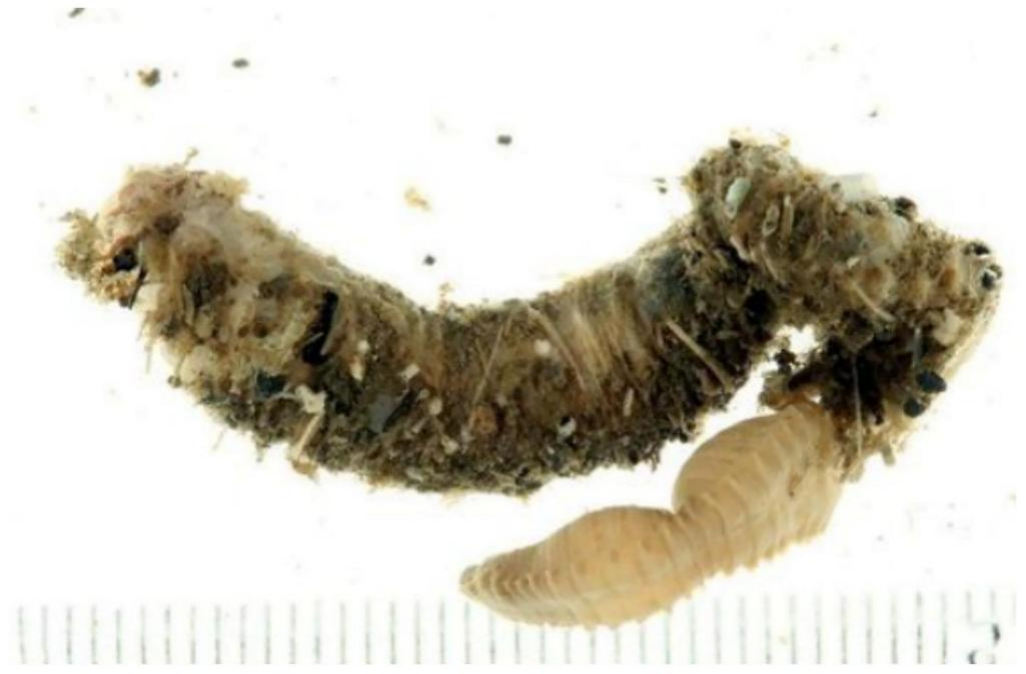
This image of *L. arborea* from GBIF Secretariat: GBIF Backbone Taxonomy https://doi.org/10.15468/39omei accessed via https://www.gbif.org/species/10614426.

Polychaeta is one of the most diverse groups within Annelida (Stiller et al. 2020), with Terebellida being a typical subgroup characterized by high species diversity. *Loimia arborea* is a ubiquitous tubular benthic organism (Garraffoni and Lana 2008). An important diagnostic characteristic of this group is the presence of uncini with pectinate teeth (Figure 1) (Londoño-Mesa and Carrera-Parra 2005; Lavesque et al. 2017; Labrune et al. 2019). Many species of Terebellida are frequently misidentified due to morphological similarities, insufficient original descriptions and the absence of type specimens (Carrerette and Nogueira 2015; Lavesque et al. 2021).

The mitochondrial genes exhibit a high degree of conservation, characterized by the presence of very short intergenic regions, the absence of introns, and maternal inheritance. These features allow the mitochondrial genome to offer valuable insights into population history over relatively short time frames (Simon et al. 2006; Yuan et al. 2015). Consequently, mitochondrial genomes have widely emerged as valuable in the field of molecular ecology, evolutionary biology, and phylogenetics (Li et al. 2017; Nitsch et al. 2024). So far, more than 10,000 polychaeta species have been described (Han and Lin 2007), but only 96 species of polychaeta mitochondrial genomes have been reported based on the data from NCBI. At present, few complete mitochondrial genomes have been used for phylogenetic analysis in Terebellida. This scarcity of foundational data has challenged the systematics of this group (Hutchings et al. 2024).

To elucidate the phylogenetic relationships of Terebellida, we assembled the first mitochondrial genome from *L. arborea* and inferred a phylogenetic of Terebellida, which will provide foundational information for further study.

## Materials and methods

The studied specimen of *L. arborea* was collected from Yellow River delta area (119.086976426E, 37.862663840N). The specimens are deposited at Nanjing Institute of Environmental Sciences, Ministry of Ecology and Environment of China, Nanjing, China (contact e-mail: muy@eastern-himalaya.cn).

Total genomic DNA was extracted from body tissue using the QIAGEN Genomic-tip kit (Hilden, Germany). The DNA sample was then subjected to next-generation sequencing at Illumina NovaSeq 6000 platform (San Diego, CA) (Cui et al. 2009; Dierckxsens et al. 2017), and assembly was performed using SPAdes (Weigert and Bleidorn 2016), by which generated 200-bp paired-end reads and a coverage depth map (Figure S1). Overlaps and redundant parts were removed to complete the full circular mitogenome. Annotation of the mitochondrial genome was carried out using the web server of MITOS (http://mitos.bioinf.uni-lzig.de/index.py) (Table S1) (Bernt et al. 2013). To revise the classification of *L. arborea*, we preliminarily identified the morphological features and then used *COX1* barcode to identify accurately. Then, we submitted the whole mitogenome to GenBank (Accession No. NC_088773.1). The mitogenome map was illustrated using the CGView (https://proksee.ca/) (Grant et al. 2023).

To explore the phylogenetic relationships, we selected the newly sequenced mitogenome of *L. arborea* and the complete mitogenomes of 13 other species from six families and *Marenzelleria neglecta* worm, which belongs to the Polychaeta, Spionida serving as the outgroup. Sequence alignment was executed using MUSCLE within MEGA 11 (Castresana 2000; Tamura et al. 2021). The best-fit model GTR + F + I + G4 was identified using PartitionFinder in PhyloSuite v1.2.2 (Zhang et al. 2020). Phylogenetic reconstruction was conducted by the maximum-likelihood (ML) by IQ-TREE with 1,000,000 replications. The tree was sampled every 1000 generations and 25% of the resulting trees were discarded as burn-in when the split frequency was lower than 0.01 (Stamatakis 2006; Nguyen et al. 2015). Phylogenetic tree was completed using by FigTree v1.4.4 (http://tree.bio.ed.ac.uk/software/figtree/) (Hu et al. 2023).

## Results

**Figure 2.**
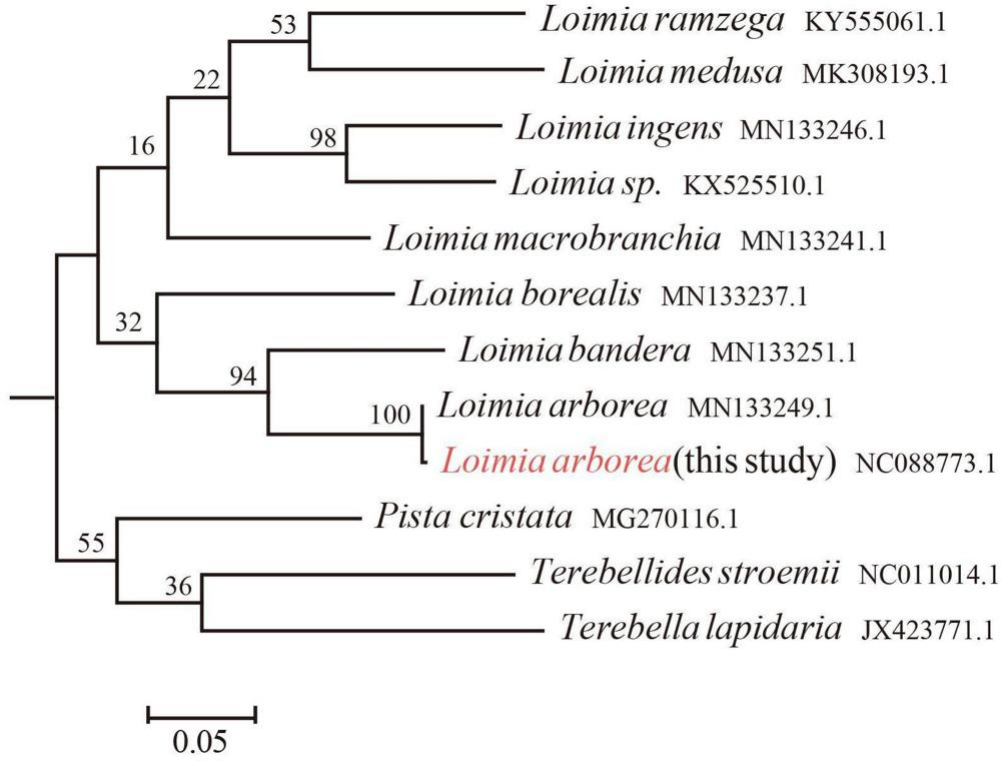
This image is a phylogenetic tree of *COX1* sequences from 12 species under the Terebellidae, in order to help us accurately identify the species.

**Figure 3.**
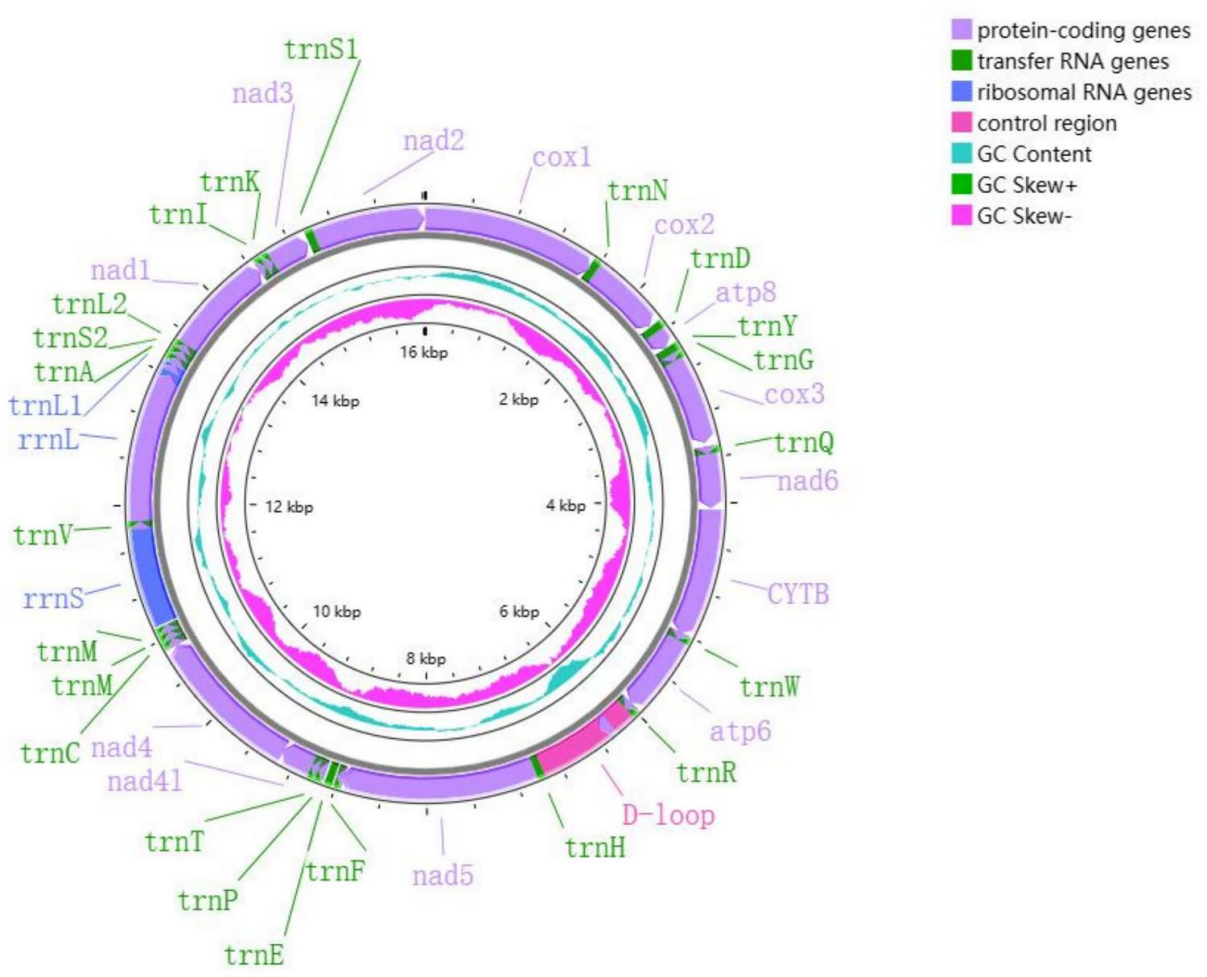
The complete mitogenome of *L. arborea*. The innermost and middle circles depict the CG skew and CG content. The outermost circle indicates the arrangements of genes from the reverse strand, with PCGs in purple, rRNAs in blue, and tRNAs in green.

The classification of *L. arborea* is accurately determined based on the *COX1* barcode (Figure 2). The mitochondrial genome of *L. arborea* reveals a typical gene composition, including 13 protein-coding genes (PCGs), 23 transfer RNA (tRNA) genes, two rRNA genes, and a putative control region (D-loop) measuring 914 bp, which includes a duplication of the *trnM* gene (Figure 3). The heavy strand (H-strand) contains all mitochondrial genes, with most of the 13 PCGs with the standard ATG codon. Variations in termination codons include complete TAA (*COX2*, *ATP8*, *COX3*, *ND6*, *ND4L*, and *ND3*), TAG (*CYTB*, *ATP6*, *ND4*, and *ND1*), and an incomplete T (*COX1*, *ND5*, and *ND2*). *ND5* is the longest PCG for length 1720 bp, while *ATP8* is the shortest for length 165 bp. This mitogenome is 16,023 bp, characterized by a nucleotide composition of 27.6% A, 20% C, 13.2% G, and 39% T.

**Figure 4.**
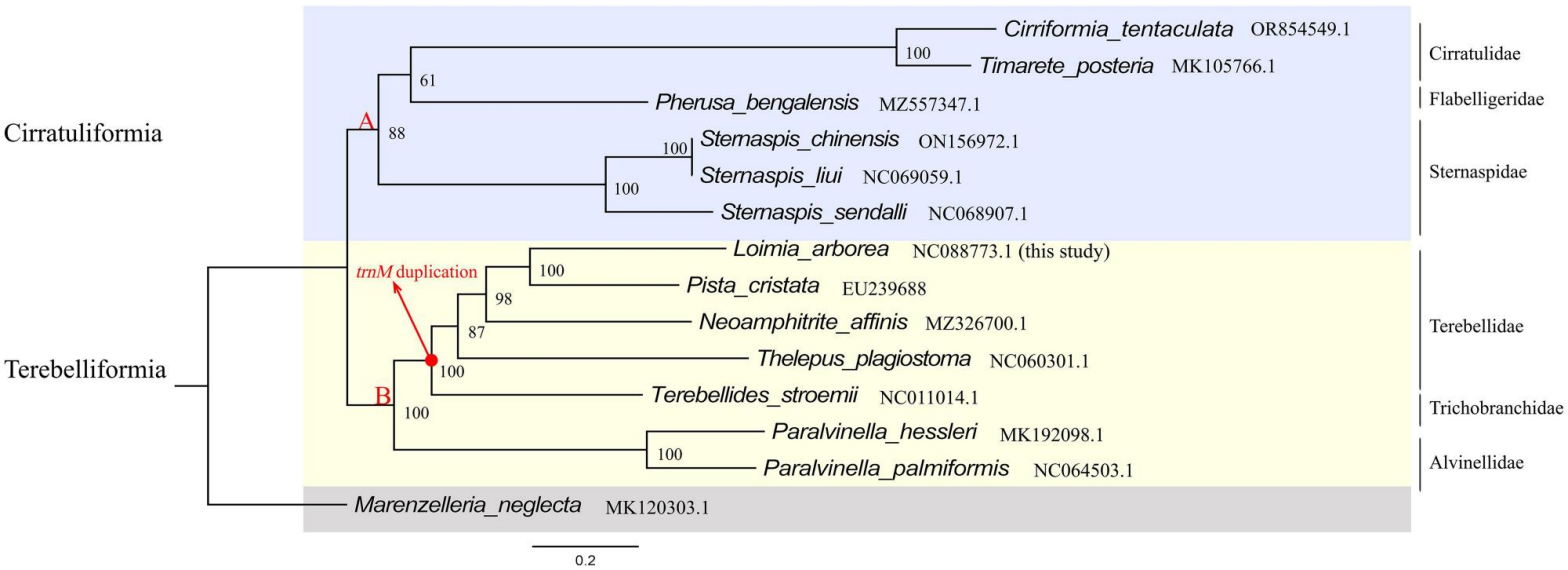
Maximum-likelihood phylogenetic trees inferred from the nucleotide sequence data of mitogenomic 13 PCGs and 1000 bootstrap replicates. The following sequences were used: *Loimia arborea* – NC088773.1 (unpublished), *Paralvinella palmiformis* – NC064503.1 (Perez et al. 2022), *Paralvinella hessleri* – MK192098.1 (Wang et al. 2019), *Cirriformia tentaculata* – OR854549.1 (Choi et al. 2017), *Timarete posteria* – MK105766.1 (Kim et al. 2019), *Pherusa bengalensis* – MZ557347.1 (Liu 2021), *Marenzelleria neglecta* – MK120303.1 (Gastineau et al. 2019), *Sternaspis chinensis* – ON156972.1 (Ge 2022a), *Sternaspis liui* – NC069059 (Ge 2022b), *Sternaspis sendalli* – NC068907.1 (Ge 2022c), *Neoamphitrite affinis* – MZ326700.1 (Nam et al. 2022), *Pista cristata* – EU239688 (Zhong et al. 2008), *Thelepus plagiostoma* – NC060301.1 (Nam et al. 2021), and *Terebellides stroemii* – NC011014.1 (Zhong et al. 2008). The numbers at the nodes indicate the maximum-likelihood bootstrap support. Clade A is for Cirratuliformia and clade B is for Terebelliformia. The red arrows and circles represent the replication of *trnM* in the presence of species from this node.

The phylogenetic relationship of *L. arborea* with 13 species was reconstructed based on 13 PCGs using ML analysis. The phylogenetic tree indicates that these species exhibit two well-supported main clades (Figure 4, clades A, B). The clade A contains Cirratulidae, Flabelligeridae, and Sternaspidae (Figure 4); clade B contains Terebellidae, Trichobranchidae, and Alvinellidae. All nodes of the phylogenetic tree exhibited bootstrap values above 85, and only one branch had lower value of 61. *L. arborea* and *Pista cristata* formed a strongly supported subclade (bootstrap = 100), indicating that they are more genetically close than the other species within clade B.

## Discussion and conclusions

It usually has been confused when we identify the species information based on morphological traits. Thus, we further confirmed the species classification based on the *COX1* phylogenetic tree with the high credible bootstrap value (BS = 100). The result strongly supported that our species is *L. arborea*.

Typical metazoan mitogenome possesses a set of 22 *tRNA* genes (Hwang and Kim 1999; Podsiadlowski et al. 2008). However, the mitochondrial genome of *L. arborea* does not conform to this pattern. Our study found that Terebellidae and Trichobranchidae both have *trnM* duplication. This duplication of methionine tRNA genes may enhance the efficiency of translation and improve adaptation to environmental changes (Weigert and Bleidorn 2016). On the other hand, the two *trnM* genes are located adjacently, which may represent a synapomorphy of Terebellidae and Trichobranchidae (Zhong et al. 2008).

The taxonomic classification of Terebellida within the Polychaeta biodiversity group has always been a topic (Garraffoni and Lana 2008; Stiller et al. 2020). Garraffoni and Lana (2008) initially classified Terebellida into two suborders based on morphological characteristics: Cirratuliformia and Terebelliformia. This classification has been supported by subsequent molecular studies, including those conducted by Struck et al. (2011). Our results further support this division with strong bootstrap support, which showed that Terebellida clustered into two main distinct clades, i.e. Cirratuliformia (clade A) and Terebelliformia (clade B), both of which exhibit (Figure 4). In our analyses, we identified the Trichobranchidae within clade B as a sister group to the Terebellidae, which corroborates findings from previous research (Garraffoni and Lana 2008; Stiller et al. 2020; Nam et al. 2022). Furthermore, our results affirm the monophyly of Terebellidae, consistent with earlier morphological investigations (Garraffoni and Lana 2008). Notably, we discovered that the duplication of the mitochondrial gene *trnM* within Terebellida originates monophyletically and serves as a synapomorphy for both Terebellidae and Trichobranchidae (Figure 4) (Garraffoni and Lana 2008; Stiller et al. 2020).

Overall, our phylogenetic support is high and the results are credible. However, in our phylogenetic results, one node exhibited low support at 61, which may be the insufficiency of species information and a minor gap in sequence alignment. Further in-depth researches are required in the future.

## Supplementary Material

Ethical approval.pdf

Table S1.pdf

Figure S1.jpg

## Data Availability

The data that support the findings of this study are openly available in GenBank at http://www.ncbi.nlm.nih.gov/, under the accession number NC_088773.1. The associated BioProject, SRA, and Bio-Sample numbers are PRJNA1100012, SRR28677105, and SAMN40962468, respectively.
