# Supplementary material for: The first complete mitochondrial genome of Loimia arborea (Polychaeta: Terebellidae) and phylogenetic analysis
Source: Mitochondrial DNA B Resour. 2024 Nov 25;9(11):1606–10. doi: 10.1080/23802359.2024.2429639 (PMC11600547; doi:10.1080/23802359.2024.2429639)
Supplement: Ethical approval.pdf [file TMDN_A_2429639_SM7683.pdf]

大理大学项目申报

动物实验伦理审查意见

我校__沐远__，现申请申报云南省高校洱海流域生态环境质量检测工程研究中心项目，项目名称为虾虎鱼科的系统发生和低氧耐受遗传机制研究（DXDGCZX03），该项目涉及虾虎鱼、鳑鲏鱼、麦穗鱼、银鱼、鲫鱼等常见鱼类及底栖大型无脊椎动物，项目负责人根据动物伦理相关要求提交动物伦理保障承诺，经大理大学实验动物伦理委员会审查，认为该项目符合动物实验伦理要求。同意申报。

大理大学实验动物伦理委员会

2022 年 11 月 21 日

大理大学实验动物伦理委员会 5329002030425
