# Supplementary material for: The first complete mitochondrial genome of Loimia arborea (Polychaeta: Terebellidae) and phylogenetic analysis
Source: Mitochondrial DNA B Resour. 2024 Nov 25;9(11):1606–10. doi: 10.1080/23802359.2024.2429639 (PMC11600547; doi:10.1080/23802359.2024.2429639)
Supplement: Table S1.pdf [file TMDN_A_2429639_SM7682.pdf]

Name	Start	Stop	Length	Spacer (+) or overlap (-)	Start codon	End codon	Strand
cox1	1	1528	1528	15	ATG	T	H
trnN(aac)	1544	1614	71	0			H
cox2	1615	2298	684	11	ATG	TAA	H
trnD(gac)	2310	2376	67	1			H
atp8	2378	2542	165	3	ATG	TAA	H
trnY(tac)	2546	2612	67	2			H
trnG(gga)	2615	2681	67	0			H
cox3	2682	3461	780	35	ATG	TAA	H
trnQ(caa)	3497	3563	67	0			H
nad6	3564	4046	483	18	ATG	TAA	H
cob	4065	5204	1140	-2	ATG	TAG	H
trnW(tga)	5203	5268	66	0			H
atp6	5269	5973	705	-1	ATG	TAG	H
trnR(cga)	5973	6042	70	0			H
D-loop	6043	6956	914	0			
trnH(cac)	6957	7021	65	1			H
nad5	7023	8742	1720	6	ATG	T	H
trnF(ttc)	8749	8815	67	18			H
trnE(gaa)	8834	8899	66	21			H
trnP(cca)	8921	8989	69	0			H
trnT(aca)	8990	9056	67	3			H
nad4l	9060	9347	288	-7	ATG	TAA	H
nad4	9341	10702	1362	-2	ATG	TAG	H
trnC(tgc)	10701	10767	67	2			H
trnM(atg)	10770	10834	65	4			H
trnM(atg)	10839	10903	65	20			H
rrnS	10924	11789	866	1			H
trnV(gta)	11791	11860	70	-10			H
rrnL	11851	13239	1389	2			H
trnL1(cta)	13242	13307	66	6			H
trnA(gca)	13314	13379	66	0			H
trnS2(tca)	13380	13447	68	0			H
trnL2(tta)	13448	13512	65	0			H
nad1	13513	14448	936	-1	ATG	TAG	H
trnI(atc)	14448	14514	67	6			H
trnK(aaa)	14521	14587	67	0			H
nad3	14588	14941	354	13	ATG	TAA	H
trnS1(aga)	14955	15025	71	-1			H
nad2	15025	16018	994	5	ATG	T	H
