# Supplementary figures and images for: The first complete mitochondrial genome of Loimia arborea (Polychaeta: Terebellidae) and phylogenetic analysis
Source: Mitochondrial DNA B Resour. 2024 Nov 25;9(11):1606–10. doi: 10.1080/23802359.2024.2429639 (PMC11600547; doi:10.1080/23802359.2024.2429639)

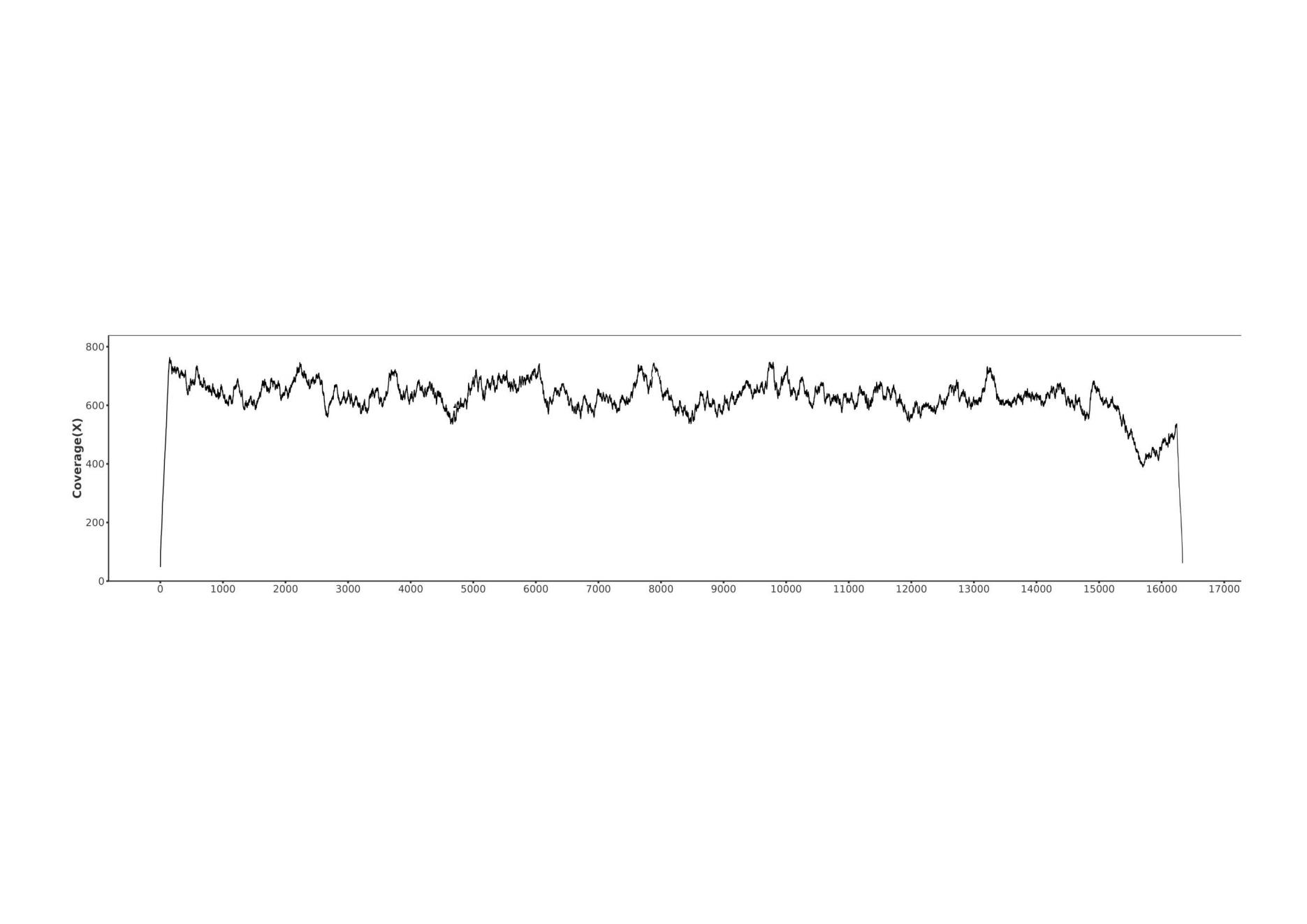

Supplement: Figure S1.jpg [file TMDN_A_2429639_SM7681.jpg]
